# Ten Lessons for Good Practice for the INHERIT Triple Win: Health, Equity, and Environmental Sustainability

**DOI:** 10.3390/ijerph16224546

**Published:** 2019-11-17

**Authors:** Ruth Bell, Matluba Khan, Maria Romeo-Velilla, Ingrid Stegeman, Alba Godfrey, Timothy Taylor, George Morris, Brigit Staatsen, Nina van der Vliet, Hanneke Kruize, Kirsti Sarheim Anthun, Monica Lillefjell, Geir Arild Espnes, Aline Chiabai, Silvestre García de Jalón, Sonia Quiroga, Pablo Martinez-Juarez, Vojtěch Máca, Iva Zvěřinová, Milan Ščasný, Sibila Marques, Daniela Craveiro, Joyce Westerink, Hanne Spelt, Pania Karnaki, Rosa Strube, Anne-Sophie Merritt, Marita Friberg, Nathalie Bélorgey, Marjolijn Vos, Dragan Gjorgjev, Inese Upelniece, Caroline Costongs

**Affiliations:** 1 Institute of Health Equity, UCL, London WC1E 7HB, UK; matluba.khan@ucl.ac.uk; 2 EuroHealthNet, 1000 Brussels, Belgium; m.romeo-velilla@eurohealthnet.eu (M.R.-V.); i.stegeman@eurohealthnet.eu (I.S.); a.godfrey@eurohealthnet.eu (A.G.); c.costongs@eurohealthnet.eu (C.C.); 3 European Centre for Environment and Human Health, University of Exeter Medical School, Truro TR1 3HD, UK; timothy.j.taylor@exeter.ac.uk (T.T.); geomorris55@hotmail.co.uk (G.M.); 4 National Institute for Public Health and the Environment (RIVM), Centre for Sustainability, Environment and Health, 3720 BA Bilthoven, The Netherlands; brigit.staatsen@rivm.nl (B.S.); nina.van.der.vliet@rivm.nl (N.v.d.V.); hanneke.kruize@rivm.nl (H.K.); 5 Department of Neuromedicine and Movement Science, Norwegian University of Science and Technology, 7030 Trondheim, Norway; kirsti.anthun@ntnu.no (K.S.A.); monica.lillefjell@ntnu.no (M.L.); 6 Department of Public Health and Nursing, NTNU Center for Health Promotion Research, Norwegian University of Science and Technology, 7030 Trondheim, Norway; geir.arild.espnes@ntnu.no; 7 Basque Centre for Climate Change, Biscaya, 48004 Pais Vasco, Spain; aline.chiabai@bc3research.org (A.C.); silvestre.garciadejalon@bc3research.org (S.G.d.J.); 8 Department of Economics, Universidad de Alcalá, 28801 Alcalá, Spain; sonia.quiroga@uah.es (S.Q.); p.martinez-juarez@exeter.ac.uk (P.M.-J.); 9 Health Economics Group, University of Exeter Medical School, Exeter EX1 2LU, UK; 10 Environment Centre (CUNI), Charles University, 162 00 Prague, Czech Republic; vojtech.maca@czp.cuni.cz (V.M.); iva.zverinova@czp.cuni.cz (I.Z.); milan.scasny@czp.cuni.cz (M.Š.); 11 Instituto Universitário de Lisboa (ISCTE-IUL), CIS-IUL, 1649-026 Lisboa, Portugal; sibila.marques@iscte-iul.pt (S.M.); daniela.craveiro@gmail.com (D.C.); 12 Philips Research, Brain, Behavior and Cognition Group, 5656 AE Eindhoven, The Netherlands; joyce.westerink@philips.com (J.W.); hanne.spelt@philips.com (H.S.); 13 Human-Technology Interaction Group, Eindhoven University of Technology (TUe), 5612 AE Eindhoven, The Netherlands; 14 Prolepsis Institute, 151 25 Athens, Greece; p.karnaki@prolepsis.gr; 15 Collaborating Centre on Sustainable Consumption and Production, 42107 Wuppertal, Germany; rosa.strube@scp-centre.org; 16 Public Health Agency of Sweden, Solna, 171 82, Sweden; anne-sophie.merritt@folkhalsomyndigheten.se (A.-S.M.); marita.friberg@folkhalsomyndigheten.se (M.F.); 17 Federal Centre for Health Education, (BZgA) Cologne, 50825 Cologne, Germany; nathalie.belorgey@bzga.de; 18 Department of Marketing, Innovation and Organisation, University of Ghent, 9000 Ghent, Belgium; marjolijn.vos@ugent.be; 19 The Institute of Public Health of the Republic of North Macedonia (IJZRM), 1000 Skopje, North Macedonia; dgjorgjev@gmail.com; 20 Riga City Council, 1010 Riga, Latvia; inese.upelniece@riga.lv

**Keywords:** policy and practice, health, equity, environmental sustainability, behaviour change, urban settings, co-creation, living, moving, consuming

## Abstract

The world’s challenges of climate change, damage to ecosystems, and social and health inequalities require changes in human behaviours at every level of organisation, among governments, business, communities, and individuals. An important question is how behaviour change can be enabled and supported at the scale and speed required. The research reported in this paper describes important lessons for good practice in changing contexts to modify behaviours for a triple win for health, equity and environmental sustainability. Authors synthesised learning from qualitative, quantitative and cost benefit evaluations of 15 case studies conducted in 12 countries in Europe. The case studies address ways of living (green spaces and energy efficient housing), moving (active transport) and consuming (healthy and sustainable diets) that support the triple win. Ten lessons for good practice were identified. These include bringing a triple win mindset to policy and practice in planning interventions, with potential to improve environmental sustainability, health and equity at the same time. The lessons for good practice are intended to support governmental and non-governmental actors, practitioners and researchers planning to work across sectors to achieve mutual benefits for health and environmental sustainability and in particular to benefit poorer and more socio-economically disadvantaged groups.

## 1. Introduction

Climate change and environmental degradation pose unprecedented threats to human health and wellbeing. All too often, poorer communities suffer the most from the widespread consequences of these problems, and benefit the least from measures taken to address them. 

The challenges of climate change, damage to ecosystems, and social and health inequalities require changes in human behaviours at every level of organisation, among governments, business, communities, and individuals [1]. An important question is how behaviour change can be enabled and supported at the scale and speed required. 

Aligning with the imperatives for action to address these challenges, the EU HORIZON 2020 INHERIT project (2016–2019) focused on identifying policies and practices that contribute to a triple win, in terms of improvements to health, equity and environmental sustainability, by creating conditions that enable people to adopt more sustainable behaviours in ways of living, moving and consuming [2]. 

Within the INHERIT project, we evaluated 15 case studies based on intersectoral interventions with potential to deliver the triple win. Based on these evaluations, this paper synthesises lessons learned about how to create an effective intervention that can create the conditions to enable behaviour change to promote a triple win, with the aim of informing governmental and non-governmental actors, practitioners, and researchers that are interested in designing such interventions.

The 15 case studies were developed based on different interventions which might deliver ‘triple win’ solutions by enabling behaviour change in the domains of living (with a focus on green space and energy efficient housing), moving (with a focus on active transport, specifically cycling and walking) and consuming (with a focus on consumption of a healthy and sustainable diet). These interventions provide examples of measures that can be implemented to contribute to achieving the triple win. Many of these promising practices represent efforts made by, inter alia, citizens, schools, community-based organisations, non-governmental organisations, small enterprises and municipalities. 

Learning from the INHERIT cases studies is useful and relevant not only to the scaling up of actions, where feasible, but also to transferring promising actions to new contexts and introducing further innovative approaches towards a healthier, more equitable and sustainable future for Europe. Indeed, an individual action multiplied across society, stimulated by many small initiatives as well as national policy tools, can shift social norms. Behavioural changes at a social scale in the way we live, move and consume are needed if societies are to become healthier, more equitable and live in a more sustainable way.

## 2. Materials and Methods 

The lessons for good practice were developed using an iterative methodology, based on learning from the implementation [3] and evaluations of INHERIT’s 15 case studies [4,5,6]. Table 1 shows 15 INHERIT case studies.

This section describes how the 15 case studies were selected, how they were evaluated, with reference to the theoretical framework provided by the INHERIT model, and how the INHERIT partners identified the ten lessons for good practice. 

### 2.1. Selection of INHERIT’s 15 Case Studies

We first carried out a literature review [7], and developed a conceptual model to guide the INHERIT project’s analytical approach. Next we collected information about 96 initiatives (promising practices) around Europe to create a database of promising practices [8]. Each INHERIT partner proposed several promising practices based on a set of criteria, including that the practices are inter-sector policies and interventions, that they address key environmental stressors, and foster the conditions that support healthy and environmentally sustainable lifestyles and behaviours, although specific evaluations may be lacking. In addition, the promising practices should be in INHERIT’s domains of interest, living (green space and energy efficient housing), moving (active transport), and consuming (healthy and sustainable food).

From the database of promising practices, INHERIT partners identified 15 case studies to be selected as INHERIT studies that would undergo qualitative, quantitative and/or cost benefit evaluations. Table 1 provides a summary of INHERIT’s 15 case studies. The 15 INHERIT case studies were selected based on a set of criteria developed by the INHERIT partners [3]. These criteria are summarized here: (i) the chosen INHERIT case studies could be implemented in the INHERIT project’s timeframe and budget; (ii) the case studies should be knowledge based, have an underlying theory of change that could be linked to the INHERIT model, and be amenable to scientifically sound evaluations; (iii) the case studies should target or have an impact on people facing socioeconomic disadvantages, and can potentially contribute to a triple win; (iv) the case studies should involve cooperation across sectors, and involve user groups; (v) the case studies should be geographically spread around Europe, and thematically spread across the areas of living, moving and consuming.

The 15 case studies were of different types and implemented at different scales. Some were relatively small scale initiatives implemented by INHERIT partners, for example, Place Standard piloted the Place Standard tool in new contexts (Latvia and North Macedonia). Others studied large-scale interventions that were already being implemented, for example, STOEMP is part of Ghent en Garde, a city wide policy promoting health and sustainable food, and Sustainable food in public nursery schools, which is city policy in Madrid. One case study combined elements of two promising practices (Gardening with Green Gym and Meat Free Monday) to create an innovative school based intervention. Details about the different types of case studies are reported elsewhere [3]. 

### 2.2. Evaluation of INHERIT’s 15 Case Studies

**Figure 1:**
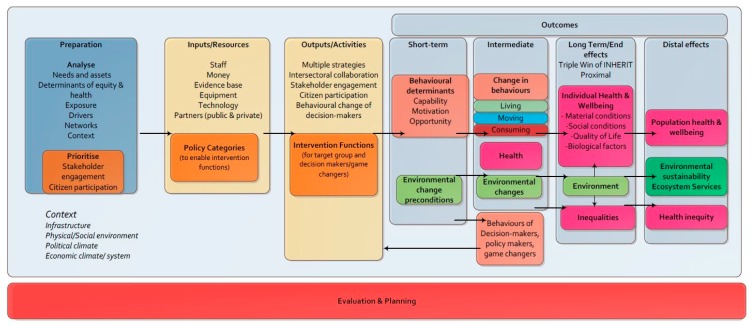
The INHERIT generic logic model.

In order to evaluate the diverse case studies in a coherent way, INHERIT partners developed a generic INHERIT logic model (Figure 1), to integrate with the INHERIT model [9]. The logic model represents the theory of how an intervention produces its outcomes. INHERIT partners used the generic INHERIT logic model to develop specific logic models for each of the case studies. The logic models were used as planning tools, which enabled partners to identify key aspects of the intervention in preparation for the case study. These included: the kinds of inputs and resources required to enable the intervention functions (staff, money, evidence base, equipment, technology, partners); expected outputs and activities (including multiple strategies, intersectoral cooperation, stakeholder engagement, citizen participation, behavioural change of policy-makers); and outcomes. The generic logic model describes outcomes and proposed indicators to be assessed in four temporal divisions: (1) Short-term outcomes: behavioural determinants in the domains of capability (e.g., building knowledge and awareness), motivation (intention to do something), opportunity (changes to the environment that enable people to change behaviours). (2) Intermediate-term outcomes: changes in behaviours, health and wellbeing, environmental change, and behaviours of decision-/policy-makers and influencers. (3) Long-term or end outcomes: impacts on health and wellbeing, quality of life, material conditions, social conditions, environment and inequalities. (4) Distal effects: impacts on population health and wellbeing, environmental sustainability and health inequity. 

Qualitative evaluations of intersectoral cooperation were carried out on 12 of the 15 case studies to investigate success factors, barriers, and the future of intersectoral cooperation in each case. For each of the 12 case studies, partners conducted one focus group, comprising partners cooperating in developing and implementing the case studies. The methodology used in the qualitative evaluation was informed by Appreciative Inquiry, an asset based approach based on questions such as what works well, and how to do more of what works well in the future [4]. 

Quantitative evaluations of nine out of the 15 INHERIT case studies were conducted to investigate their impacts on the targeted populations [5]. These evaluations were based upon the INHERIT model [9], which integrates theory about the environmental determinants of health and health inequalities with behaviour change theory [10]. Quantitative methods included two randomised control studies (using pre-post design and questionnaire survey), several quasi-experimental studies, cross-sectional questionnaire surveys related to the case studies, a questionnaire survey representative for five European countries [11] and observations of use and activity level. Quantitative methods were complemented by qualitative studies with target groups in some case studies. 

Given the complexities involved in measuring health outcomes, particularly given the short amount of time to implement/evaluate the INHERIT interventions, we did not expect to see measurable changes in health outcomes. Therefore, the focus of the evaluations was on short term and intermediate outcomes. Quantitative evaluations in some case studies examined how interventions might influence people’s capabilities, opportunities and motivation to change their behaviours. Most evaluations assessed health related behaviours (e.g. physical activity or aspects of healthy eating depending on the nature of the intervention). Some cases studies assessed well-being or life satisfaction. INHERIT partners developed an evaluation framework, which identified from the literature validated and reliable tools to assess physical activity, dietary behaviours, and well being, as well as demographic and socioeconomic indicators [5]. Partners used these tools where relevant to their case studies, or identified others by searching the literature. 

Cost benefit analyses (CBA) were conducted on four case studies [6]. The CBA quantified in monetary terms social costs and benefits of these interventions, in order to find out their social desirability, following economic efficiency criteria. Short-term and future medium-term impacts were accounted for, using appropriate ranges of discount rates to derive present value equivalents. A participatory methodology was used in the case of Thinking Fadura, using citizens’ surveys and stakeholders’ workshops to identify which items should be evaluated as societal impacts, as well as which further intangible (not monetized) impacts should be taken into account.

### 2.3. Synthesis of Learning from INHERIT’s Case Studies 

INHERIT partners identified and synthesized lessons learned from the implementation and evaluations of the case studies using an iterative and deliberative methodology. To facilitate discussions about what the main elements of good practice are, we grouped the INHERIT case studies by the following themes:

- Community-based initiatives around food. Case studies: De Voedseltuin, PROVE, STOEMP.
- School-based initiatives. Case studies: GemüseAckerdemie, Gardening with Green Gym and Meat Free Monday, Sustainable Food in Public Schools.
- Open/green space initiatives. Case studies: Malvik Path, Restructuring Green Spaces, Restructuring Residential Outdoor Areas, Thinking Fadura.
- Energy efficiency in homes. Case studies: Eco Inclusion, Retrospective Analysis of Energy Efficient Investments.
- E-coaching applications around moving. Case studies: UrbanCyclers (active transport) and Lifestyle-coaching (physical activity).
- Participatory governance approaches towards the triple win. Case study: Place Standard.

Short summaries of all the case studies were prepared that described the target groups, what inspired the creation of the initiative, success factors, outcomes and impact, and ideas for future development. Then we conducted an expert workshop in Brussels, to review the compiled evidence and draw conclusions about what can be considered as overarching lessons for good practice. This workshop brought together 10 experts from a wide range of backgrounds (including public health, environmental research, social psychology, environment and health economics, policy and epidemiology) from across the INHERIT consortium. Before the workshop, each participant read the selected reports. During the workshop, all participants familiarised themselves with at least two of the case studies. Participants were assigned into two groups. One group discussed findings from the implementation report on the 15 INHERIT case studies [3] and the qualitative evaluations [4] that focused on intersectoral cooperation in implementing the case studies. The second group deliberated on implications of the findings from the impact evaluations [5] and the cost benefit analyses [6], both of which included qualitative and quantitative methodologies. The findings from the two group discussions were then fed back into plenary discussions for further deliberation. Subsequently, the lead author (R.B.) prepared a draft report detailing the identified lessons for good practice, based on deliberations during the expert workshop on the evidence from the implementation and evaluations of the case studies. The draft report was circulated to a wider group of INHERIT researchers (including all co-authors) for review and feedback. The 10 lessons for good practice were validated by the INHERIT project steering group at an INHERIT consortium meeting, with minor amendments.

## 3. Findings

**Figure 2:**
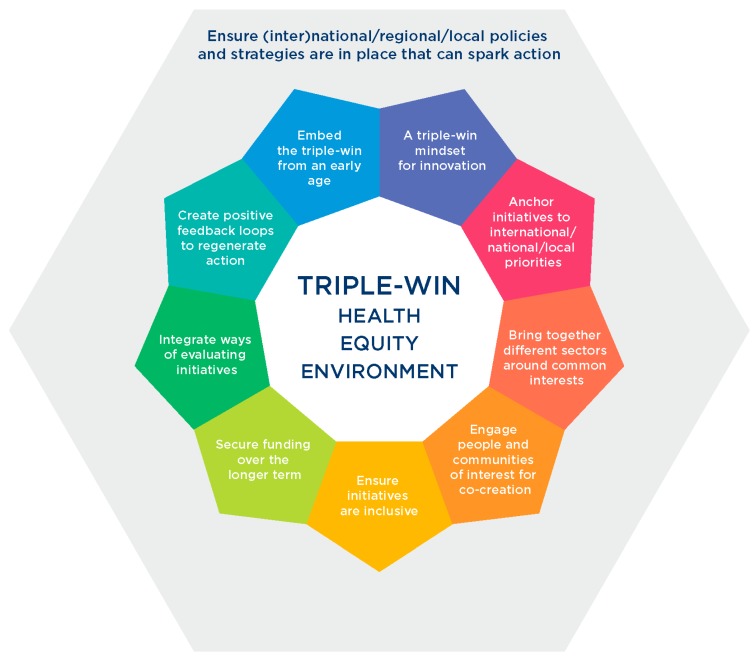
Overarching lessons for good practice for the INHERIT triple win.

Here we describe the overarching lessons for good practice (Figure 2), identified from evaluations of INHERIT’s 15 case studies, all of which have potential to modify behaviours of policymakers, the private sector and citizens by changing the contexts in which people live, move and consume. 

### 3.1. A Triple Win Mindset for Innovation

We identified a triple win mindset as crucial in taking an approach that breaks down traditional sectoral silos and enables innovation in intersectoral cooperation. A triple win mindset means setting out with the intention of creating synergies across sectors to create a triple win for health, equity and environmental sustainability. 

All INHERIT case studies are considered to be triple win initiatives. Yet the potential for a triple win was not explicit in the original objectives of all interventions. Often, it was their engagement in INHERIT that made them recognise their potential to have multiple societal benefits. Taking as an example that might traditionally be thought of as a health intervention, the activity tracker and associated smart phone application used in the Lifestyle e-coaching case study can exist as a stand-alone system to support individuals in tracking their activity levels. Yet bringing a triple win mindset to this case study opens out wider questions such as: is the technology effective among all social groups and those with low activity level (the basis of the INHERIT research on this case study), and, if so, how can the technology be made more widely available? Taking the environment into account leads to further questions, for example: are there usable parks and safe areas for people to be more active near their homes? Such questions contribute to systems thinking that is fundamental to create conditions that enable behavioural change for the triple win.

As another example, the Place Standard Tool was designed to guide discussions about what needs to be targeted to improve a place [12], but applying it with a triple win mindset in North Macedonia identified different key indicators (air quality, waste, water quality) for possible inclusion. This would further develop the Place Standard Tool as a tool to guide thinking and planning for a triple win for health, equity and environmental sustainability. 

Triple win thinking, and the awareness of those engaged that they are contributing to the health and well-being of community members as well as to broader ambitions related to the urgent climate crisis, can strengthen the commitment and motivation for action of those involved. 

Bringing a triple win mindset to the table demands creative thinking and discussions to plan the necessary steps that can bring different sectors together to make a triple win happen. Yet, as evidenced in several INHERIT case studies, impacts of interventions may not always be unambiguously positive, and there may be trade-offs as well as synergies across sectors. For example, as noted in the case of Sustainable Food in Public Nursery Schools, organic food production in the area local to Madrid is not at the scale needed to supply Madrid’s public nursery schools, therefore a trade-off needs to be made between providing organic food and the environmental cost of transport. 

In the case of Energy Efficient Investments, while the environmental benefits are likely to be positive due to energy and carbon savings, the targeting of lower socioeconomic groups and those in social housing for energy efficiency investments may exacerbate health inequalities, unless the measures put in place are appropriately designed to avoid the sealing of properties and the negative health impacts associated with this [6,7,13,14]. Identification of trade-offs and adverse consequences, as well as potential positive impacts, gives extra weight to the imperative for applying a triple win mindset to a broad range of initiatives such as those investigated in the INHERIT project. 

### 3.2. Ensure (inter)National/Regional/Local Strategies are in Place that can Spark Action

The presence of (inter)national/regional/local strategies for improving health inequalities, and environmental sustainability creates a supportive environment for local action. The UN Sustainable Development Goals [15] are a useful framework to work within because they set out an internationally agreed direction for development that requires action across different sectors towards multiple goals, including health, equity and environmental sustainability goals. In this context, European, national, and local strategies can provide enabling and supportive environments in which the kinds of initiatives that are needed to achieve these goals can flourish. For example, the STOEMP network is part of Ghent en Garde, the municipality’s response to the Milan Urban Food Policy Pact (MUFP) [16] which aims to develop sustainable food systems and healthy diets for citizens [17]. 

However, governments can do more to provide a facilitating policy environment in which small scale initiatives can thrive. Individuals and organisations have a role in advocating for the kinds of international, regional, national and local strategies that stimulate or regulate actions needed for a triple win. In turn, governments at all levels can facilitate the broad stakeholder engagement necessary to develop policies and strategies to deliver the triple win through local initiatives.

In this context, impacts of school-based initiatives such as Gardening with Green Gyms and Meat free Monday in the UK would be boosted if outdoor learning were to be institutionalized by integrating it within the mandatory national curriculum. Furthermore, national public procurement regulations could be used to support healthier and more sustainable food in schools. This would support the shift to more sustainable farming practices, and consumption of less meat and more plant-based food, which has been called for by the International Panel on Climate Change.

### 3.3. Anchor Initiative to International/National/Local Priorities 

Anchoring initiatives to international, national and local priorities can help embed local level initiatives in a whole systems approach that is necessary to address complex challenges. This point is linked to ‘Ensure (inter)national/regional/local strategies are in place that can spark action’ (Section 3.2 above). We include both because one enables the other. So while these two lessons are linked, they are separate. Individuals and communities wishing to develop local level action for a triple win can generate greater momentum by linking the initiative to higher level priorities of improving health, reducing inequalities and promoting environmental sustainability. This depends on the existence of (inter)national/regional/local policies and strategies that enable triple win initiatives. International, national and local priorities help support powerful arguments that can bring together diverse sectors around common interests. For example, an idea for a new initiative, such as a food garden in a disadvantaged area in Ghent, can gain traction by arguing that it contributes to the aims of STOEMP in reaching disadvantaged groups, the overall Ghent en Garde objectives, and the MUFP for healthier and more sustainable food. As another example, in the context of England, where the prevalence of child obesity (ages 10/11) was 20% in 2017/18 [18], the National Childhood Obesity Strategy recognises that schools have a fundamental part to play in supporting healthy lifestyles for children [19]; this provided an anchor for the Gardening with Green Gym and Meat free Monday pilot initiative. Nevertheless, enabling this practice to be maintained and potentially scaled up nationally would require integration of outdoor learning in the national curriculum, as was raised above (Section 3.2).

### 3.4. Bring Together Different Sectors around Common Interests

Bringing together different sectors around common interests is a further key element in creating triple win initiatives. This is demonstrated by INHERIT’s case studies in the context of developing green and open spaces, and in sustainable food initiatives, such as STOEMP and Sustainable Food in Nursery Public Schools. An effective approach in developing interventions requires both horizontal collaboration, between groups with more or less equal power, as well as vertical linkages between those with differing levels of power (which can include anchoring in higher level priorities). 

The importance of multisectoral action and intersectoral cooperation to achieve common goals is widely accepted but may be difficult to do in practice. Common interest regarding local challenges was a pronounced theme in INHERIT initiatives and is related to personal commitment. One of the success factors was a common goal shared among people from different sectors. 

While common interest can bring people together, lack of coordination can affect the success of the intervention. Bringing people together at an early stage to identify areas of common interest and to develop common goals is a crucial part of the process. However, it can take time to build up the necessary rapport and trust between stakeholders to facilitate cooperation; trust must be nurtured as a valuable resource. 

In the INHERIT team’s experience, the process of using the INHERIT model [9] as a tool in stakeholder workshops has proved highly influential in opening up discussions among stakeholders about the opportunities for multiple benefits of initiatives of various kinds. The INHERIT model combines environmental impact assessment with health impact assessment and equity impact assessment in a way that enables people and stakeholders from multiple sectors to identify potential impacts and risks for any particular initiative. The use of an adapted form [20] of the INHERIT model to inform the participatory methodology and, through this, to derive a set of potential positive and negative impacts for cost benefit analysis in the Thinking Fadura initiative provides a good example of how this way of thinking can contribute to cooperation across sectors. It is crucial that this step is carried out before going on to think about how potential impacts and risks can be quantified or evaluated.

The INHERIT model as a tool for health, environment and equity impact assessment provides a way of identifying potential positive as well as adverse impacts, and therefore diverges from standard regulatory environmental impact assessments used, for example, in infrastructure development projects, and designed to identify environmental risks to health.

### 3.5. Engage People and Communities of Interest for Co-creation

Engaging people and communities with the mindset of co-creation should be a central theme in triple win initiatives. This is important because people need to be involved in the decisions that affect their lives and living environment. Being involved in decisions that affect one’s life is fundamental to participatory governance models at the local level that aim to ensure social inclusion. In addition, the opportunity to be involved in decisions that affect one’s life is a core element of empowerment. This has been positioned by the WHO Commission on Social Determinants of Health as being key to enabling people to live healthy and flourishing lives [21]. In addition, being involved in decision-making gives people a vested interest in the success of the initiatives and encourages active participation in a way that creates a positive feedback loop. We see that in INHERIT initiatives such as Restructuring Green Space and Malvik Path, where involving people in a meaningful way in planning and developing processes creates places that local people actually use in ways that improve their lives and creates more engaged residents who want to protect and enhance the places they use. 

We also see that it is not always easy to engage people. INHERIT has developed some fundamental requirements in this respect, and proposes possible solutions. It is important to engage the community from the inception of an intervention and engagement should be continued throughout the process. Co-creation is also important for securing a sense of ownership among participants, but that will only happen when the participation is meaningful and not tokenistic. The level of success of co-creation depends on addressing the actual needs of residents and the extent to which communities are engaged in processes of design and implementation. How much the community participates also depends on creating rapport and trust [22]. It takes a long time to build trust and a very short time to lose it.

Every kind of community engagement requires some kind of commitment from those involved. The extent of commitment varies along a sliding scale, from attending meetings to share opinions to volunteering labour, as, for example, in De Voedseltuin (Food Garden). It is important, then, that those wishing to engage residents provide a good rationale for people to be engaged. Incentives can vary from providing refreshments and a welcoming environment at meetings to providing more official recognition of roles. Local campaigns, news coverage, and events (as in Malvik Path) that help create an identity can raise the profile of initiatives and give recognition to engaged communities. Furthermore, such campaigns may provide sources of information that inform people about what is happening and how they can become involved and give them confidence to join new initiatives. In some cases, there may be a need to develop participatory skills in the communities, for instance through educational programmes. 

Several INHERIT case studies have worked with volunteers based on the notion that volunteers are crucial for maintaining the project longer term. However, recruiting volunteers can be challenging. Some practices found it difficult to recruit volunteers, as in the case of the GemüseAckerdemie where volunteers were needed in the daytime during the week. Furthermore, recruitment of volunteers from across the social spectrum can be difficult where the benefits of volunteering are not explicit. It is important to avoid the act of volunteering being a luxury, affordable only by the more advantaged in society. Some initiatives are intrinsically beneficial for volunteers, for example De Voedseltuin where volunteers receive training and produce from the food garden, and for others, there are benefits of volunteering that might not be apparent or visible in the short term, such as self esteem and enhanced wellbeing [23], and health/environmental literacy. However, the association between volunteering and wellbeing may not always be causal [24].

Supporting volunteers includes developing infrastructure that facilitates capacity-building through knowledge exchanges. For example, training can be video recorded and widely distributed, online courses can be developed, and existing teaching programmes for volunteers can be replicated. Volunteers are sometimes involved in peer delivered interventions in public health [25], and in other kinds of peer training too, such as the operating model of Eco Inclusion. Further scaling-up or transferring of the peer training model to other areas should be informed by existing peer training models developed in the area of public health [26]. Experiences from INHERIT show that training that helps volunteers develop transferable life skills is beneficial [7]. For example, De Voedseltuin offers workshops to volunteers that also help them reintegrate into the job market.

### 3.6. Ensure Initiatives are Inclusive

Initiatives should be inclusive so that everyone can potentially benefit. This aligns with the concept of ‘proportional universalism’ used in discussing how to combat social inequalities in health; it means actions or initiatives should benefit everyone across the social scale and be deployed at a scale and intensity proportionate to the level of disadvantage [27].

Therefore, to gain the greatest benefits, interventions for the INHERIT triple win may need to take into account the specific needs of groups facing different disadvantages (e.g. older people, refugees), which are not homogeneous in themselves, so differentiated action may be needed. For example, it is important to pay attention to age, gender and cultural diversity and sensitivity when implementing and evaluating interventions. This was demonstrated to good effect in the Restructuring Green Space case study in which an underused green space was restructured, using inclusive participatory methods, into an attractive green space widely used by all members of a diverse community.

INHERIT case studies showed that interventions that have a positive impact on health and the environment which take a settings approach, such as schools and green spaces, are effective ways of also addressing health equity. More targeted approaches that focus on specific groups facing disadvantages may also be needed, as reflected in the STOEMP case study, and Eco Inclusion. In such cases, it is crucial not to stereotype or stigmatise people, as this can place an additional burden on people suffering from relative deprivation.

### 3.7. Secure Funding over the Longer Term

Secure funding over the longer term was identified as necessary, and challenging. Of course, no initiative can operate in the absence of funding, from inception through to maintenance and sustainability. In this regard, government funding for local initiatives is vital, and should be built in to national and local government strategies linked with achieving the SDGs, climate change mitigation and adaptation, population health, social inclusion, and environmental sustainability.

That being said, local initiatives that gain funding from national or local government schemes are at risk in the event that other funding priorities emerge. Alternative funding sources should be explored. One solution for financial sustainability of community-based initiatives might be, therefore, to have a hybrid business model, with diverse funding partners, including public, private and collective sources. Another funding solution is to merge the funding for environmental and social initiatives, recognising that there are mutual environmental and social benefits.

In addition to funding, there is a need for adaptation of legislation or policy frameworks in such a way that it facilitates local intersectoral initiatives and co-creation processes. In the case of Restructuring Residential Outdoor Areas, the property owner received 50% of the restructuring costs from a fund set up by the Swedish government to support upgrades of outdoor areas in socially deprived residential areas in a way that involved multiple stakeholders, including residents.

Cost benefit analyses can support decisions regarding investing in initiatives. However, while these analyses are useful, they should not be the only basis for decision-making, bearing in mind that not all potential benefits and risks can be quantified, and that some perceived impacts might not be beneficial across the social spectrum, or distributed evenly across various population segments [7]. As demonstrated in the case of Thinking Fadura, cost benefit analyses can provide an intersectoral engagement process to help identify potential positive impacts as well as adverse consequences of initiatives, which can be valuable inputs to planning and development.

### 3.8. Integrate Ways of Evaluating Initiatives

Evaluation of initiatives is all too often seen as an optional extra, or something that is done as an academic exercise. INHERIT’s experience is that evaluation of initiatives not only helps to understand the processes of implementation, intersectoral cooperation, impacts and benefits, but also to learn about what could be done better to build synergies across sectors and to enhance outcomes. Evaluations are both summative and formative. 

Evaluation of projects and programmes in real world settings is challenging and time-consuming. The evaluation methodology can be improved in a scenario with more time and resources. For example, in PROVE a combination of longitudinal quantitative and qualitative data collections, following consumers after baseline measurements, would allow better understanding of how PROVE influences behaviour, critical moments for change, or even the interplay of different determinants at the different stages of behaviour change. 

Researchers also need to take into account the demands made by the research on stakeholders and survey respondents. It is important to take time to engage stakeholders, and to explain the purpose and potential benefits of the research to participants invited to respond to surveys. 

Despite the inherent challenges of real world evaluations, they are highly valuable to project and programme managers, not least because they bring out important issues for reflection that can influence further developments and improvements. To enable this to happen, it is important that researchers report results of evaluations to project partners and stakeholders. Indeed, PROVE partners and implementers have already developed an idea to extend the PROVE initiative to schools, which may help to promote consumption of fruit and vegetables across a wider range of social groups.

Long-term evaluation plans should be in place with plans for maintenance and sustainability of each initiative. Interventions that might be successful in the short term might not work out longer term; again, short-term evaluation might not yield measurable outcomes. INHERIT’s quantitative evaluations [5] were, by necessity, given the funding period of the project, based on short-term evaluations, but our experience suggests that it is important to conduct long-term evaluation. The duration of the evaluation will need to vary from case to case, depending on the particular health outcomes being addressed, and to reflect the extent to which the impacts of any intervention may change over time. 

### 3.9. Create Postive Feedback Loops to Regenerate Action

INHERIT case-studies can create positive feedback loops that can regenerate action or stimulate further action. A case in point is UrbanCyclers which supports more commuter cycling on the roads. More urban cycling creates incentives for cities to improve cycling infrastructure, which encourages more commuter cycling, creating a positive feedback loop. Similarly, having an activity tracker, as in the case of Lifestyle e-coaching, and being motivated to walk more, might encourage people to seek out green space and tree-lined roads. Building a need among people for more green space and more pleasant environments for cycling and walking should be an incentive for municipalities to give higher priority to restructuring areas to create more usable green spaces. Again, as discussed above, engaging people and communities in planning and development of settings-based interventions, as in Malvik Path and Restructuring Green Space, as well as more broadly in applying the Place Standard Tool, can reinforce the value of participating in community development processes and create a sense of ownership. It is important, however, to demonstrate to people and communities that their engagement is worthwhile and results in tangible changes to settings that improves lives.

### 3.10. Embed the Triple Win from an Early Age

Giving children the best start in life has been identified as crucial for improving health and reducing health inequalities across the life course [27]. Healthy behaviour at a young age will provide health benefits later in life [7]. For example, a relationship between active travel behaviour during childhood and walking during adulthood has been observed [28], suggesting that policies to encourage active transport among children could result in benefits not only during childhood but also throughout the life cycle of the individual. 

Several INHERIT case studies demonstrate how it is possible to embed the triple win at an early age by working with children in school-based or community based settings. Involving children and young people in such initiatives is important in creating positive feedback loops across generations as a way of embedding environmental sustainability, intergenerational equity and future population health. Experiences in early life lay the foundation for future attitudes to eating a healthy diet and being physically active, for respecting and protecting the natural environment and for cooperative social behaviour [7]. Additional societal benefits accrue because children influence their parents’ and peers’ attitudes and behaviours. Therefore, initiatives that involve children and young people in school and community settings can have long term benefits towards the triple win. 

The involvement of parents in a school based programme is also perceived as being important by teachers [29]. In the case of Gardening with Green Gyms and Meat free Monday, there are several ways to involve parents, such as sending newsletters, providing homework tasks for parents and children and involving them in the maintenance of gardening during holidays [30]. There is evidence that linking with the wider community is a success factor for school-based interventions [31] and local organisations can take care of the school garden out of school hours [29]

Links between school and community can go both ways. Discussion among stakeholders can provide opportunities to achieve an extra win if they consider children and young people. For example, in discussions about PROVE, partners and stakeholders discussed the possibility of piloting a subscription to PROVE in a school, which would simultaneously widen the market reach of PROVE farmers, increase availability of fresh local fruit and vegetables to children in a school setting, and make the initiative more socially inclusive.

## 4. Discussion

This paper has identified lessons for good practice to support actions intended to change the contexts and conditions in which people live, move and consume in order to enable behaviour changes in ways that contribute to a triple win for health, equity and environmental sustainability. Despite extensive studies in the areas of living, consuming, and moving separately [7], to our knowledge, these aspects have not been investigated together before under an overarching common analytical framework. 

The key lessons derived from INHERIT case studies distil essential elements from heterogeneous practices undertaken in different countries across Europe, at different levels, involving different actors, and using different interventions. This diversity should ensure that these key lessons capture the most relevant elements that were identified either over multiple contexts, actors, policy levels, INHERIT domains or by using different evaluation methods.

All INHERIT case studies enable behaviours that have the potential to improve health, equity and environmental sustainability. It is worth emphasising that in evaluating complex programmes in real life settings it is necessary to gather information from different sources with both quantitative and qualitative methodologies. At the same time, it is necessary to understand the limitations imposed by complexity on the level of certainty that can be achieved in undertaking evaluations of real-world activities.

Several limitations have been identified that might impact the findings used to synthesise the lessons for good practice. The selection of case studies was affected by the project timeframe and the available budget for their evaluation. The case studies were at different scales, in different contexts, and involved a range of methodological approaches. Some case studies were evaluated in more detail than others. In some cases, the limited duration of the intervention meant that results were inconclusive. This indicates the need for longer intervention periods with long-term evaluation plans and multiple follow-ups. In addition, the work in INHERIT shows the challenges inherent in quantifying all the potential impacts of case studies, and demonstrates the importance of working within the INHERIT conceptual model and grounding evaluations in the wider literature.

Clearly, more research is needed to build on INHERIT’s findings. In particular, it is important to understand the implications for health equity of triple win interventions. These we have only been able to assess in largely qualitative terms, based on expert judgment. Longer term funding for triple win interventions is needed, with evaluation embedded alongside to help monitor the impacts over time of these. In addition, it is important to identify potential hurdles and bottlenecks in transferability and scaling up of triple win practices to different contexts and circumstances.

Furthermore, focusing on behaviour change of the business sector (e.g., food industry) and working towards new business models may help to realize the triple win.

Overall, INHERIT’s research contributes to the case for investing in interventions for a triple win, since even small steps can lead to significant impacts. Indeed, localisation, meaning local control and implementation of interventions, is vital if countries are to transition to sustainability. 

## 5. Conclusions

This paper synthesises lessons for good practice in developing interventions intended to enable people to adopt more sustainable behaviours in ways of living, moving and consuming, and thereby contribute to a triple win for health, equity and environmental sustainability. These findings contribute to and align with wider discussions about the kinds of synergistic approaches needed to improve population health and address climate change [32]. They also contribute to discussions that are gaining pace in the face of growing alarm about the environmental crisis about how to shift away from economies based primarily on the growth imperative to economies focusing on well-being [33]. Such discussions also reflect a growing awareness of the synergies between climate change mitigation policies and other societal goals that contribute to well-being, including health, education, jobs, environmental quality, and social inclusion [34].

There is, for example, a large and expanding literature that reports how green space contributes to well-being, and how creating more readily accessible and good quality green space can provide opportunities to socialise and be active, and at the same contribute to climate change mitigation by providing protection against high temperature, and buffering the effect of heavy rainfall [7,35].

At the heart of these discussions about societal goals is the need to shift to integrated rather than ‘sector based’ objectives and approaches to achieve multiple objectives and to mainstream concepts of ‘fairness’ and redistribution. This means reframing measures of progress and reassessing and re-focusing policy priorities.

The INHERIT project has contributed to this by spearheading an innovative approach that intended to disrupt traditional disciplinary boundaries, developing a broad evaluation framework, based on the INHERIT model. It also provides a unique contribution through its study of a range of local initiatives that aim to deliver multiple societal objectives. Localised action can be a powerful force of change, since it gives people ownership and provides them with direct experience of the benefits of the initiative, which can motivate them to press for the maintenance and expansion of such actions. It is also clear, however, that cohesive and coherent policies at higher levels of government to support, spread and scale such ‘triple win’ actions are needed. Experience with and evidence on the multiple benefits of such actions, like that gathered through INHERIT, can help to achieve this.

## Figures and Tables

**Table 1:** Summary descriptions of the 15 INHERIT case studies.

Name of Case Study, Country	Nature of Case Study	Type of Evaluation
The Food Garden (De Voedseltuin), the Netherlands	An urban community gardening initiative in a disadvantaged area	Qualitative
PROVE, Portugal	Sustainable farming practices creating closer links among producers and consumers	Qualitative, Quantitative
STOEMP (within Ghent en Garde policy), Belgium	Local food initiatives for healthier and more sustainable food	Qualitative
Gemüse Ackerdemie (Vegetable Academy), Germany	Increasing the number of volunteers to support vegetable academy programs for school aged children to connect with nature and origins of food	Qualitative
Gardening with Green Gym and Meat Free Monday, United Kingdom	Gardening activities with children in a primary school and promotion of a meat free day/week	Qualitative, Quantitative
Sustainable food in public nursery schools, Spain	Introducing sustainable foods in public nurseries in Madrid	Qualitative, CBA
Malvik Path, Norway	Reconstruction of a disused railway track into a recreational path connecting two communities	Quantitative, CBA
Restructuring Residential Outdoor Areas, Sweden	Regeneration of and improved access to an open space	Qualitative, Quantitative
Restructuring Green Space, the Netherlands	Regeneration of an open green space in a housing estate in a disadvantaged area	Qualitative, Quantitative
Thinking Fadura, Spain	Providing access to previously private green spaces to the general public	Quantitative, CBA
Eco-inclusion, Germany	Capacity building and awareness program among migrants about energy efficiency in housing	Qualitative, Quantitative
Retrospective Analysis of Energy Efficiency Investment, United Kingdom	Energy efficiency investments including double-glazing, insulation and improved heating systems	CBA
Lifestyle e-coaching, the Netherlands and Greece	A lifestyle e-coaching application including a physical activity tracker and smartphone application	Quantitative
UrbanCyclers (now known as Cyclers), Czech Republic	A smartphone application to promote regular cycling in cities	Qualitative, Quantitative
Place Standard, Latvia and North Macedonia	Implementation of Place Standard Tool: a framework to structure conversations about place and community	Qualitative
